# Visualizing diversity: the Oregon Health & Science University Educational Use Photo Diversity Repository

**DOI:** 10.5195/jmla.2021.1171

**Published:** 2021-07-01

**Authors:** Pamela Pierce, Linda Felver

**Affiliations:** 1 piercepa@ohsu.edu, Digital Scholarship and Repository Librarian, Oregon Health & Science University, Portland, OR; 2 felverl@ohsu.edu, Associate Professor, OHSU School of Nursing, Oregon Health & Science University, Portland, OR

**Keywords:** diversity, inclusion, cultural humility, health science education

## Abstract

**Background:**

Health science education needs images that represent both the diversity of patients served and the diversity of its students and clinicians. To begin to address this need, a nurse educator and librarian collaborated to launch the Oregon Health & Science University (OHSU) Educational Use Photo Diversity Repository. This online resource provides educators of health professional students with access to photos of pathophysiological conditions in skin of various colors so their students can increase their awareness of issues related to health and diversity and prepare themselves for more effective clinical work with their future patients.

**Case Presentation:**

The OHSU Educational Use Photo Diversity Repository became a university-wide project, leveraging the thoughts of an image advisory board, clinicians, faculty members, administrators, and students. Key considerations were given to the workflows used to submit photos as well as the controlled vocabulary for submitting images. The repository was started with photos already in existence, with future plans to have images taken specifically for the repository.

**Conclusions:**

This repository is playing an important role as OHSU and the health sciences in general reflect on the role of systemic racism in health care and clinical education. Negotiating issues of consent, patient health information, and privacy around using different technologies to take photos is a complex and ongoing process. The repository provides opportunities for closely examining these processes and creating improvements that result in more equitable education.

## BACKGROUND

As cultural humility education in health care is important for reducing health disparities, educators should understand their institution's approach to issues of race within the curriculum [1] [2]. Different from cultural competence, cultural humility is a recognition of the role of power in health care settings [3]. Cultural humility also emphasizes self-reflection and active listening [4]. Changing demographics make this even more important. It is estimated that by 2060, people of non-European descent will comprise almost half of the US population [1]. New clinicians should be prepared for this evolving reality and “equipped with the best tools to maximize patient-centered communication with a culturally, racially, and socioeconomically diverse patient population” [3, 604]. To that end, some recommendations on learning objectives and methods for teaching focus on identifying and examining bias, acknowledging disparities, and finding solutions, as well as on communication and negotiation skills [5].

While libraries have partnered to provide photo collections to their institutions through licensing resources and creating unique collections, the essential nature of the need for photographs of pathophysiological conditions that depend strongly on skin color and tone for interpretation requires a committed and sensitive partnership and educational approach. The Oregon Health & Science University (OHSU) Educational Use Photo Diversity Repository arose from this context. For example, jaundice, pallor, inflammation, and purpura caused by pathophysiological processes change the color of skin by making it potentially appear more yellow (jaundice), have less underlying red tone (pallor), become more reddish (inflammation), or have more purplish areas (purpura) [6]. The nature of color change with cyanosis, a change in skin tone resulting from poorly oxygenated blood, depends on a person's baseline skin color; white skin and mucous membranes become bluish, and dark skin may become grey or whitish around the mouth [7]. Health professional students need to learn to recognize these changes in skin of any color and tone.

Without exposure to educational content and images that build awareness of and demonstrate these diverse expressions of disease, health professional students will not develop the knowledge needed to recognize them in their patients and may end up perpetuating health care inequalities and experiences that contribute to systemic disparities. Current photo repositories often focus on specific disciplines, such as the photo gallery of Stanford Medicine's Newborn Nursery, Columbia University's Digital Reference of Ophthalmology, and the Rheumatology Image Library, which is only available to American College of Rheumatology members. All of these repositories were created with the goal of providing visual images to aid in diagnosis. The homepage for the Newborn Nursery Gallery specifically say that the photos are for “educational purpose only” [8].

Education of health professionals takes place within a larger cultural context. After the death of George Floyd, protests focusing on white supremacy and racial violence emerged, and Portland, Oregon, experienced months of protests. In a statement after the death of George Floyd, OHSU's president, Danny Jacobs, wrote, “Acknowledging the fact that racism exists is the first imperative step in committing to change. Let's educate ourselves about the historical cultural norms that are embedded into our everyday lives that contribute to bias and prejudice …” [9].

It is important to acknowledge that the students and faculty at OHSU are mostly white—69.4% of the student population was white in fall 2019 [10]. OHSU has committed to a complex evaluation of the institution so it fully reflects and embodies values of equality and inclusion. One part of this work is making sure that research projects, especially those involving public health, address racial and ethnic inequalities. OHSU also took the stance of supporting students and staff who wished to be involved in lawful civic and community activities calling for justice.

The widespread social justice movement has resulted in greater attention to the images used in health care classes. In England, a medical student published a handbook, *Mind the Gap: A Handbook of Clinical Signs in Black and Brown Skin* [11], that draws attention to the importance of making sure medical education is widely reflective of patients and students of color. A recent news article laments the lack of diverse photos in helping to diagnose COVID-19 skin rashes [12].

While the need for diverse photos is rising, attention to the ethical and technological issues surrounding these images is essential. Recommendations related to the Health Insurance Portability and Accountability Act (HIPAA) and the use of smartphones in a medical setting should include having clear, written protocols for the consent for clinical photography and a signature required for consent for every possible use of the image [13]. Apps have been experimented with as a way of gaining patient consent and ensuring secure storage and transmission of photographs. The need for photos that truly represent the reality in which health science practitioners work has never been greater.

## CASE PRESENTATION

In October 2017, Dr. Linda Felver, an associate professor of nursing with more than thirty years of teaching experience, launched the Photo Diversity Project in response to the lack of available diverse photographs for use in teaching pathophysiology and clinical pharmacology courses to nursing students.

The lack of diverse medical images for education became clear to Dr. Felver when she was searching for images of early-stage pressure injuries in dark skin. In darker skin, identification of early-stage pressure injuries involves the recognition of more subtle changes than in light skin [15]. However, when Dr. Felver's Photo Diversity Project was launched, of the thirty-four pressure injury photographs in VisualDx, a clinical decision support system, only two showed dark skin. Both of those photographs involved the most serious type of pressure injury, with obvious deep tissue damage. No photographs of the early or intermediate stages of pressure injuries in dark skin were provided.

A sequence of events elevated the idea for the project from one faculty member in the School of Nursing to a university-wide collaboration that includes the OHSU Library. After Dr. Felver presented her work at the OHSU Symposium on Educational Excellence, the chairperson of the OHSU Educators' Collaborative invited her to present at Education Grand Rounds. During the discussion portion of Dr. Felver's Education Grand Rounds presentation, the need for a source of images of visible pathophysiologies that did not use white skin as the default was emphasized. A faculty member from the School of Medicine who was present proceeded to contact the OHSU vice provost for educational improvement and innovation and explain the interest and need. Subsequently, the vice provost gathered key players from the OHSU Library and School of Medicine and invited Dr. Felver and Pamela Pierce from the OHSU Library to a meeting, where they were introduced to each other. During this meeting, it was proposed that they collaborate and write a grant proposal.

In the spring of 2019, the OHSU Library successfully applied for a $12,000 Health Sciences Library Partnership Award from the National Network of Libraries of Medicine-Pacific Northwest Region to create the OHSU Educational Use Photo Diversity Repository. The repository is set up as a collection within the digital asset management system (DAMS), which was created in 2016 to manage, share, and safeguard the university's digital assets and includes robust HIPAA and Family Educational Rights and Privacy Act (FERPA) compliance. The Educational Use Photo Diversity Repository, a distinct collection of photographs within the DAMS, improves the discoverability of images, increases the number of photos, and sets up a clear framework for how the images can be used.

## GOVERNANCE AND STAFFING OF PROJECT

Engaging campus stakeholders, including clinicians, administrators, and faculty members, was identified as essential to the success of the repository, and a shared governance approach was determined to be the best method. Early on, the repository's review committee realized that the project needed to be submitted to the university's Institutional Review Board (IRB). After reviewing the repository protocol, the IRB determined that it was not human subjects research and thus was exempt from IRB review and approval.

Consent remained essential to the project's design, as many of the subjects of these photos have been treated unethically throughout the history of medical research. For example, the Tuskegee Study, which began in 1932, intentionally left syphilis untreated in subjects. The study's occurrence related to norms within the US science community such as “disproportionally focusing on poor and disadvantaged groups for medical experimentation and demonstration” [16]. The standard OHSU photo release form was adapted for the project and put into a web-based secure Qualtrics form so that patients could consent using their phones (Appendix 1). The release form briefly described the repository as a “digital collection of photographs that teachers of health professions students can use to help their students visualize conditions in diverse populations.”

The project review committee helped us develop connections around the university that could benefit the project. We fine-tuned the process for submitting photographs, achieved buy-in from students, received feedback on how the project was described, and had students talk to faculty and clinician members within their network. In the summer of 2019, we connected with several OHSU community groups to gather feedback, including the Alliance for Visible Diversity in Science, the Middle Eastern/South Asian Association, the All-Hill Student Council, the Health Policy Interest Group, Internal Medicine Division chiefs, and School of Nursing undergraduate faculty members. Contact was made with the groups via email, and each group issued an invitation to one of their meetings. Overall, the groups, and especially their faculty members, expressed interest and excitement in the project. The Alliance for Visible Diversity in Science gave important feedback on how the project could be described more effectively. For example, they suggested the inclusion of “Educational Use” in the title of the repository to make it immediately clear how the photos would be used.

We also formed an Image Advisory Group, which includes clinicians and nonclinician faculty members. The central function of this group is to provide peer review of photographs submitted to the repository, a function not provided by the initial project review committee of the grant. Members can also advise on applying MeSH terms to the photographs and suggest places from which additional photographs can be obtained. The group organized one meeting in January 2020. Shared documents were created in Box, an online platform for file sharing and collaborative editing licensed by OHSU, enabling participants to give feedback virtually.

As of November 2020, the group has not yet provided peer review of photographs, because the current photographs were already vetted by the faculty who donated them. Members of the group have promoted the project to faculty members and clinicians on campus. The group also drafted inclusion and exclusion criteria for photographs (Table 1).

**Table 1:** Inclusion and exclusion criteria for photographs

Inclusion Criteria	Exclusion Criteria
Shows a recognizable, visible pathophysiological condition	No verification of photo release form
Involves skin of color	Reveals protected health information in the background or foreground of the photo that is not covered by the photo release form
Is clear enough for educational use	Unclear visually, making it unsuitable for educational use
Was submitted with the appropriate release form, as verified by Pamela Pierce	Reinforces an uneven power differential with a person with white skin having more power

## KEY CONSIDERATIONS AND REQUIREMENTS FOR IMPROVING DISCOVERABILITY

The images within the OHSU Educational Use Photo Diversity Repository needed to be cataloged by terms that faculty and clinicians would use when searching for diseases and symptoms. An early step was selecting a controlled vocabulary to use when cataloging images. Medical Subject Headings (MeSH) was selected as the appropriate vocabulary for this project. MeSH is a controlled and hierarchically organized vocabulary produced by the National Library of Medicine. MeSH is also familiar to the people who would be searching for photos, because it is how subject headings are assigned to articles within MEDLINE. The grant funding supported a public health student worker who contributed to the project by troubleshooting the upload process and creating metadata for the photographs.

One consideration regarding cataloging the images was whether to include skin color, race, or ethnicity in the metadata for the photos. Because race is a social construct rather than a biological one [17] and skin color and tone vary widely within any racial or ethnicity category, we decided not to catalog by race or ethnicity [18]. Furthermore, while some clinicians use Fitzpatrick Skin Type to categorize by skin color, we decided not to categorize the photographs in this way, as the Fitzpatrick Skin Type was devised by a dermatologist to rate how vulnerable a skin is to burn from ultraviolet light [19] and was not intended to be a classification for other purposes. Current discussion of the limitations of Fitzpatrick Skin Type recommends not using it to classify skin color or as a proxy for race and ethnicity [20]. Therefore, no satisfactory solution to describing skin color by dermatologists and other health professionals has yet been created, although this continues to be discussed [21].

An important part of laying the infrastructure for launching the repository was figuring out the steps for people to submit photographs. First, the workflow depended on Box Capture, an application that uploads photos and videos taken with your phone directly into Box. Box Capture ensured that patients' photos would not be stored on personal phones in violation of HIPAA. However, Box Capture is not available on Android devices. The app also crashed when people tried to invite collaborators. The upload process is included in Appendix 1 and may be streamlined in the future with additional technical expertise.

One of the most prominent challenges with the repository is that physicians, nurse practitioners, and physician assistants are busy, so attention focuses on shortening patient interactions [22]. A long and complex uploading process made it unrealistic that photos could be taken and uploaded and release forms completed within the constraints of the patient interaction. The team behind the repository strives to make it a useful educational tool that adapts successfully to the realities in the clinical setting.

Given the plan to have people take and submit photographs, the next major step was to develop a waiver and permissions process for taking photos and getting them uploaded into the repository. At OHSU, marketing and communications staff have primary responsibility for coordinating release forms, and their forms were adapted for the repository's use. A photograph release form and an authorization form for patient photos were simplified and combined into one form and reviewed by OHSU's legal counsel.

The location of the release form changed over time. We started with Box, which involved giving people access to the Box folder. However, OHSU started using Qualtrics as its approved survey platform, which eventually became the preferred storage for the release forms. Testing by staff also showed that it was relatively simple to fill out the release form on people's phones.

## EXPANDING CLINICIAN PARTICIPATION

Faculty and clinician buy-in also required more outreach, as they are most likely to have access to patients (the source of photos) and to use the repository. The biggest results from these early interactions were thinking through how to publicly describe the project and how to create initial excitement.

Our outreach plan has two essential parts. The first part is to gather more photos. One way this has occurred is by having our committee members talk about the project to campus groups like the Educator's Collaborative, a cross-school and program collective of faculty, staff, and learners who engage in the academic mission. At these meetings, representatives from different departments learn about the need for photos and how to let us know about additional images that can be considered for the repository.

Increased campus discussion on health care inequities and broad discussions around race and racial violence have given the project additional attention and opportunities for gathering photos. We are also working with a clinician to understand workflows connected to EPIC, the system in which patient records are entered and stored at OHSU. Understanding these workflows is the first step toward building a process that retrieves photos directly from EPIC, while still valuing consent and patient rights.

The second part of our outreach plan is to make sure that educators know about the project and begin using the photographs in classes. This will become easier as we acquire additional photos. One barrier to this plan is that users of the DAMS must have an account. However, once educators log into the DAMS, they automatically see the photos that they can use in their classes without having to search. Educators will be reached through virtual campus meetings as well as through wider campus marketing, including social media and *OHSU Now* news stories. Educators will also be consulted to determine additional image needs.

We expanded the collection despite challenges with uploading photos from phones by using photographs that were already in existence. The Dermatology Department had a collection of photographs that we were given permission to search for appropriate images involving people of color. The resulting 137 photos were downloaded, tagged with MeSH vocabulary, and uploaded into the Educational Use Photo Diversity Repository within the DAMS. These images can only be used for education within OHSU, as specific consent documentation for these photos is absent.

We researched OHSU's policies around photography and consent, which involved consulting with the Communications Department, the Integrity Department, and the Office of Information Privacy and Security. The determination was that patient photos that do not show any protected health information do not require HIPAA authorization. The dermatology photos are also for OHSU educational use only. A note of transparency about the lack of consent attached to the photos was added to each image.

## DISCUSSION

As of November 2020, about 100 internal educators have access to the OHSU Educational Use Photo Diversity Repository, including users in OHSU's Simulation Centers who specifically look for inclusive photos to use in patient role-plays. Additional educators will be added as they express interest and receive required training on the DAMS. Usage data are not available at this point. Training educators on accessing the repository provides an opportunity to talk about library resources, including guides recommending additional resources on health disparities and inclusion within academic medicine.

We appreciate our role in engaging educators in partnerships to continue focusing on cultural issues in health care as an essential part of education [1]. Libraries and organizations across the country have committed to a deep evaluation of the role of systemic racism within their practices. This includes STEM-focused institutions that are actively working to counteract racism [23]. Whiteness must be decentered for the library profession to move forward [24]. A core component of that evaluative process is identifying actions to take that can create positive change. The OHSU Educational Use Photo Diversity Repository is one such action. We have received support from OHSU's Center for Diversity and Inclusion as partners in this work, and we will continue to connect with them as this project evolves and is operationalized.

One piece of the commitment to cultural humility is respectful engagement of those who may or may not choose to contribute their images. Consent is foundational to the repository. Our awareness of the history of medicine shows us that the populations we seek to work with have not always been given the opportunity to consent to what is done to them within a health science framework [16]. Our own reckoning with the lack of documented consent for some of the photos included in the repository points to the importance of facing these issues in as transparent a way as possible.

The implementation of the Educational Use Photo Diversity Repository is an opportunity for providers to talk to patients about how their photos are used. It is also a chance for clinicians to make sure they are following all guidelines regarding the proper storage and processing for patient images. Initiatives like this allow many members of the health science community, including educators, clinicians, students, and librarians, to be involved in creating more equitable health care for all.

## Data Availability

There are no data associated with this article.

## SUPPLEMENTAL FILES

**Appendix 1** Process for uploading images into the Photo Diversity Repository
