# Supplementary material for: Visualizing diversity: the Oregon Health & Science University Educational Use Photo Diversity Repository
Source: J Med Libr Assoc. 2021 Jul 1;109(3):472–7. doi: 10.5195/jmla.2021.1171 (PMC8485945; doi:10.5195/jmla.2021.1171)
Supplement: Supplementary file 1 — Appendix 1 Process for uploading images into the Photo Diversity Repository [file jmla-109-3-472-s01.docx]

# Appendix 1

1. Email Pamela Pierce to get access to the photo diversity repository folder in Box.
2. Using the appropriate app store (Google Play or Apple Store), download and install the **Box Capture** mobile application onto your phone. ***Note:*** *Make sure to choose the* ***Box Capture*** *app and* ***not*** *“Box for EEM”.* This is the tool you will use to capture photos.
3. Please make sure you have a Box account. You can set one up when you log into the app. Make sure that you can see the OHSU Photo Diversity Repository Folder that you emailed about, because this is where you will store the photos you take. Below we have more about how to take a photo). If it is your first time using Box Capture, be sure to give the app permission to use your camera by tapping **Allow** when prompted. You can also use Box Capture to **scan** documents, see "[Scanning Documents](https://community.box.com/t5/How-To-Guides-for-Mobile/Using-and-Managing-Box-Capture/ta-p/4655#docscan)" below for more information.
4. Fill out photo release form. That can be found here. <https://ohsu.ca1.qualtrics.com/jfe/form/SV_bO84B9XLFnQeoAd>. It’s really easy for people to do this on their phone.

# Using Box Capture to take and save images

## **Capturing Images**

**Simplified version of these steps for review**

1. Go to the top of the screen and select photos to take an image.
2. Make sure that geolocation informed is turned off and that review is enabled.
3. Tap the circle at the bottom of the screen to take an image.
4. Move content to the OHSU Educational Use Photo Diversity Repository.

Within Box Capture, go to the top of the screen to select the type of media you want to capture, in this case Photos. Tap **Photo** to take pictures.

1. Remember if it is your first time using Box Capture, be sure to give the app permission to use your camera by tapping **Allow** when prompted.
2. When you capture photos for the Photo Diversity Repository, g**eolocation information should be OFF.** You can toggle geolocation metadata on or off from the **Settings** menu (see **"Settings"** below for more information).
3. Do NOT have your content automatically uploaded; instead, be able to review it before uploading by enabling review. Tap the **Review** slider at the bottom-right of the screen. See "[Review](https://community.box.com/t5/How-To-Guides-for-Mobile/Using-and-Managing-Box-Capture/ta-p/4655#Review)" below for more information.
4. Tap the **circle** at the bottom of the screen to capture an image.
5. Move content to folder in Box called OHSU Educational Use Photo Diversity Repository.

**Important Note:** Box Capture's review setting is disabled by default for images, meaning your content will be uploaded to Box as soon as you capture it.
